# Expression of the Kynurenine Pathway in Human Peripheral Blood Mononuclear Cells: Implications for Inflammatory and Neurodegenerative Disease

**DOI:** 10.1371/journal.pone.0131389

**Published:** 2015-06-26

**Authors:** Simon P. Jones, Nunzio F. Franco, Bianca Varney, Gayathri Sundaram, David A. Brown, Josien de Bie, Chai K. Lim, Gilles J. Guillemin, Bruce J. Brew

**Affiliations:** 1 Peter Duncan Neurosciences Research Unit, St Vincent’s Centre for Applied Medical Research, Sydney, Australia; 2 St Vincent’s Clinical School, Faculty of Medicine, UNSW, Sydney, Australia; 3 Neuroinflammation group, Faculty of Medicine and Health Sciences, Macquarie University, Sydney, Australia; 4 Department of Neurology, St Vincent’s Hospital, Sydney, Australia; University of Colorado Denver School of Medicine, UNITED STATES

## Abstract

The kynurenine pathway is a fundamental mechanism of immunosuppression and peripheral tolerance. It is increasingly recognized as playing a major role in the pathogenesis of a wide variety of inflammatory, neurodegenerative and malignant disorders. However, the temporal dynamics of kynurenine pathway activation and metabolite production in human immune cells is currently unknown. Here we report the novel use of flow cytometry, combined with ultra high-performance liquid chromatography and gas chromatography-mass spectrometry, to sensitively quantify the intracellular expression of three key kynurenine pathway enzymes and the main kynurenine pathway metabolites in a time-course study. This is the first study to show that up-regulation of indoleamine 2,3-dioxygenase (IDO-1), kynurenine 3-monoxygenase (KMO) and quinolinate phosphoribosyltransferase (QPRT) is lacking in lymphocytes treated with interferon gamma. In contrast, peripheral monocytes showed a significant elevation of kynurenine pathway enzymes and metabolites when treated with interferon gamma. Expression of IDO-1, KMO and QPRT correlated significantly with activation of the kynurenine pathway (kynurenine:tryptophan ratio), quinolinic acid concentration and production of the monocyte derived, pro-inflammatory immune response marker: neopterin. Our results also describe an original and sensitive methodological approach to quantify kynurenine pathway enzyme expression in cells. This has revealed further insights into the potential role of these enzymes in disease processes.

## Introduction

**Fig 1:**
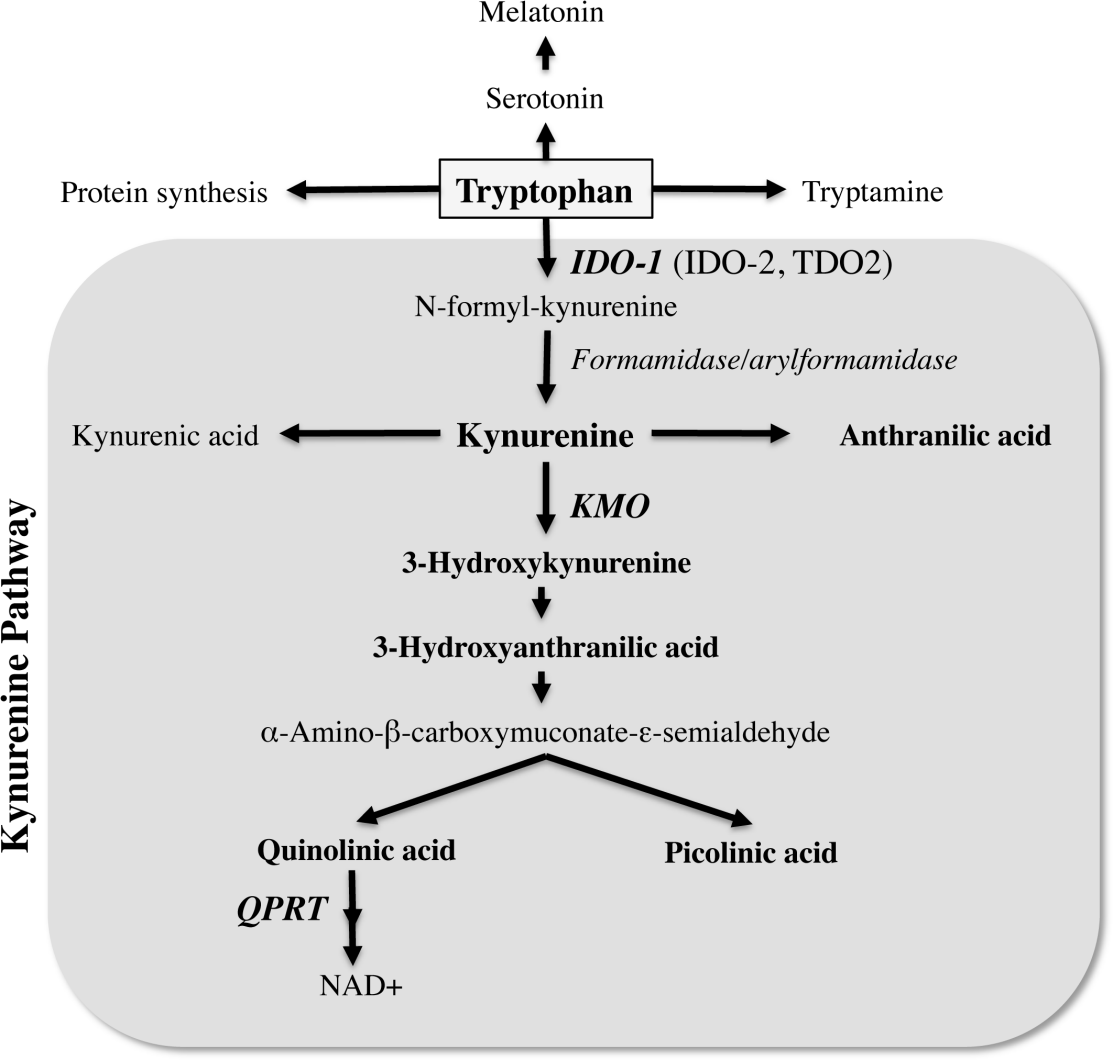
Simplified overview of the kynurenine pathway of tryptophan metabolism. The three main enzymes: indoleamine 2,3-dioxygenase (IDO-1), kynurenine 3-monooxygenase (KMO) and quinolinate phosphoribosyltransferase (QPRT) are shown abbreviated in bold italics whilst the main metabolites measured in this study are written in bold.

The kynurenine pathway (KP) is the major route for the catabolism of the essential amino acid tryptophan, which also leads to production of several potent neuroactive and immunomodulatory intermediates (Fig 1). In the initial step of the KP, tryptophan is oxidized by either tryptophan 2,3-dioxygenase (TDO2), indoleamine 2,3-dioxygenase 1 (IDO-1) or IDO-2 [1–3]. TDO2 is primarily expressed in the liver [4, 5] whilst IDO-1 is the predominant enzyme extra-hepatically. IDO-1 is expressed in numerous cell types, including macrophages, microglia, neurons and astrocytes [6–8] and can be induced by several inflammatory molecules, including lipopolysaccharides, amyloid peptides and HIV proteins [9–11]. The most potent activator of IDO-1 is interferon gamma (IFN-γ) [3, 12]. IFN-γ induces both the gene expression and enzymatic activity of IDO-1 [13, 14]. The more recently identified IDO-2 possesses similar structural and enzymatic activities to IDO-1 [2], however it is unclear whether it is functionally active in human cells [15].

In terms of KP metabolites, kynurenine (KYN) is the central intermediate formed following the breakdown of TRP to N-formyl-kynurenine by IDO-1, IDO-2 or TDO. N-formyl-kynurenine is then broken down by the action of formamidase or arylformamidase to form KYN (Fig 1). KYN is catabolised by the action of several enzymes including: kynureninase, to generate anthranilic acid (AA) and kynurenine 3-monoxygenase (KMO) to generate several other neuroactive intermediates. These include the neurotoxic and free-radical generator 3-hydroxykynurenine (3-HK) [16, 17], the free-radical generator and immunomodulatory 3-hydroxyanthranilic acid (3-HAA) [16, 18], the excitotoxin and N-methyl-D-aspartate (NMDA) receptor agonist quinolinic acid (QUIN) [19] and the neuroprotectant picolinic acid (PIC) [20] (Fig 1). Among all the KP metabolites QUIN is likely to be one of the most important in terms of biological activity and toxicity [21]. QUIN is the substrate for quinolinate phosphoribosyltransferase (QPRT), which initiates several metabolic steps and ultimately results in production of nicotinamide adenine dinucleotide (NAD^+^) [22](Fig 1).

The KP has been recognized in recent years as an important regulator of the innate and adaptive immune system [23]. IDO-1 has been shown to exert potent immunosuppressive effects in a range of contexts. These include the prevention of foetal rejection during pregnancy [24], suppression of autoimmune disease [25, 26] and expression in tumours to evade immune attack [27]. These immunosuppressive effects on T lymphocytes likely result from tryptophan depletion and the direct effects of tryptophan catabolites [28–31]. A third proposed mechanism is that KP activation modifies the properties of antigen presenting cells [32]. The finding that the enzymes involved in tryptophan metabolism are induced during dendritic cell maturation supports this hypothesis [33].

Some of the kynurenines, such as QUIN and 3-HAA, can selectively target immune cells undergoing activation, consequently suppressing T cell proliferation [34, 35]. More recently KYN has also been shown to be involved in immuno-regulation through its ligand function for the aryl hydrocarbon receptor (AhR) [36]. KP metabolites have also been shown to act in concert to produce an additive effect [37]. IDO-1 up-regulation and accelerated and sustained degradation of tryptophan represent a key indicator of inflammation. Indeed, inflammation and resulting immune activation lead to the activation of the KP and the concomitant increased production of the excitotoxin QUIN [38]. QUIN has been associated with the pathogenesis of a wide range of inflammatory diseases and neurodegenerative disorders including Alzheimer’s disease, AIDS dementia, multiple sclerosis, amyotrophic lateral sclerosis and Huntington’s disease [39–42].

Carlin *et al* initially demonstrated in 1987 that type I and type II interferons could up-regulate the expression of IDO-1 in human peripheral blood mononuclear cells [43]. In the same year, Ozaki and colleagues showed the induction of IDO-1 by interferons in human peripheral blood monocytes that had been isolated by plastic adherence [44]. This increase in IDO-1 expression by interferons could be inhibited with commonly used anti-inflammatory drugs, such as indomethacin and dexamethasone. Subsequently IDO-1 up-regulation by interferons was also shown in monocyte-derived macrophages [45, 46] and the anti-inflammatory interleukin-4 (IL-4) was found to inhibit the induction of IDO-1 mRNA and enzymatic activity in human monocytes [47]. Despite these early studies, and the potential clinical relevance of KP activation in immune cells, the characterization of KP enzyme expression and metabolite production remains poorly defined in lymphocytes and monocytes. There are no data on human lymphocyte KP expression and the time course for KP products in PBMCs has not been delineated. This is important to establish if there is a role for KP expression in lymphocytes or whether the effects of KP activation are mediated solely by monocytes. Can the KP be up regulated in lymphocytes in an inflammatory environment or does this immune regulation rely on monocytes? Examining the expression of KP enzymes over a time course is crucial to establish if the KP is activated sequentially. As outlined above, the KP produces cytotoxic and/or cytoprotective metabolites, depending on the stage of activation. This has important implications for cellular damage or protection if at different time-points distinct arms of the KP predominate. This is given further significance in light of recent findings that some KP products antagonize each other, along with the potential toxic effects of sustained KP activation.

Activation of the KP in cells has conventionally been measured by analysis of metabolite production in culture supernatants, typically using techniques such as UHPLC and GC-MS. Expression of KP enzymes has been limited to analysis at the mRNA level or qualitative measurement using western blot. In order to gain quantitative data at a cellular level on the expression of key enzymes in the KP, we have optimized and used intracellular flow cytometry staining. We describe here for the first time the expression of IDO-1, KMO and QPRT in human peripheral blood mononuclear cells (PBMCs) untreated (control) or treated with varying doses of IFN-γ over different time-points.

## Materials and Methods

### Peripheral blood mononuclear cell isolation

PBMCs were isolated from leukopacks (buffy coats) obtained from the Australian Red Cross blood service under material supply agreement 13-10NSW-10. Buffy coats were diluted 1:2 in phosphate-buffered saline (PBS), carefully layered on top of density gradient media (Isolymph, GE Healthcare) and centrifuged at 500 x g for 30 minutes at 4°C. The PBMC layer was removed, washed in PBS then counted on a haemocytometer using trypan blue exclusion and cryopreserved (^-^196°C) at a concentration of 20 x 10^6^ cells/mL in fetal bovine serum (FBS) containing 10% dimethyl sulfoxide (DMSO). PBMC were thawed as required for experiments and their viability assessed by trypan blue exclusion (consistently >90% viable cells).

### Peripheral blood mononuclear cell culture

PBMCs were seeded in 12 well plates at a concentration of 1 x 10^6^ cells/mL in Aim V serum free media (Life Technologies). PBMCs were treated with increasing doses (0, 50, 100, 500, 1000 IU/mL) of IFN-γ (Miltenyi Biotec) for 24, 48 or 72 hours. Cell free supernatants were removed for KP metabolite quantification by HPLC and GC-MS and stored at ^-^80°C. PBMCs were harvested from wells, with a 5 minute incubation with 0.05% Trypsin/EDTA (Life Technologies) used to detach all adherent monocytic cells. PBMCs were then washed in PBS and fixed in 3.7% paraformaldehyde for 10 minutes at 4°C.

### Flow cytometry

**Fig 2:**
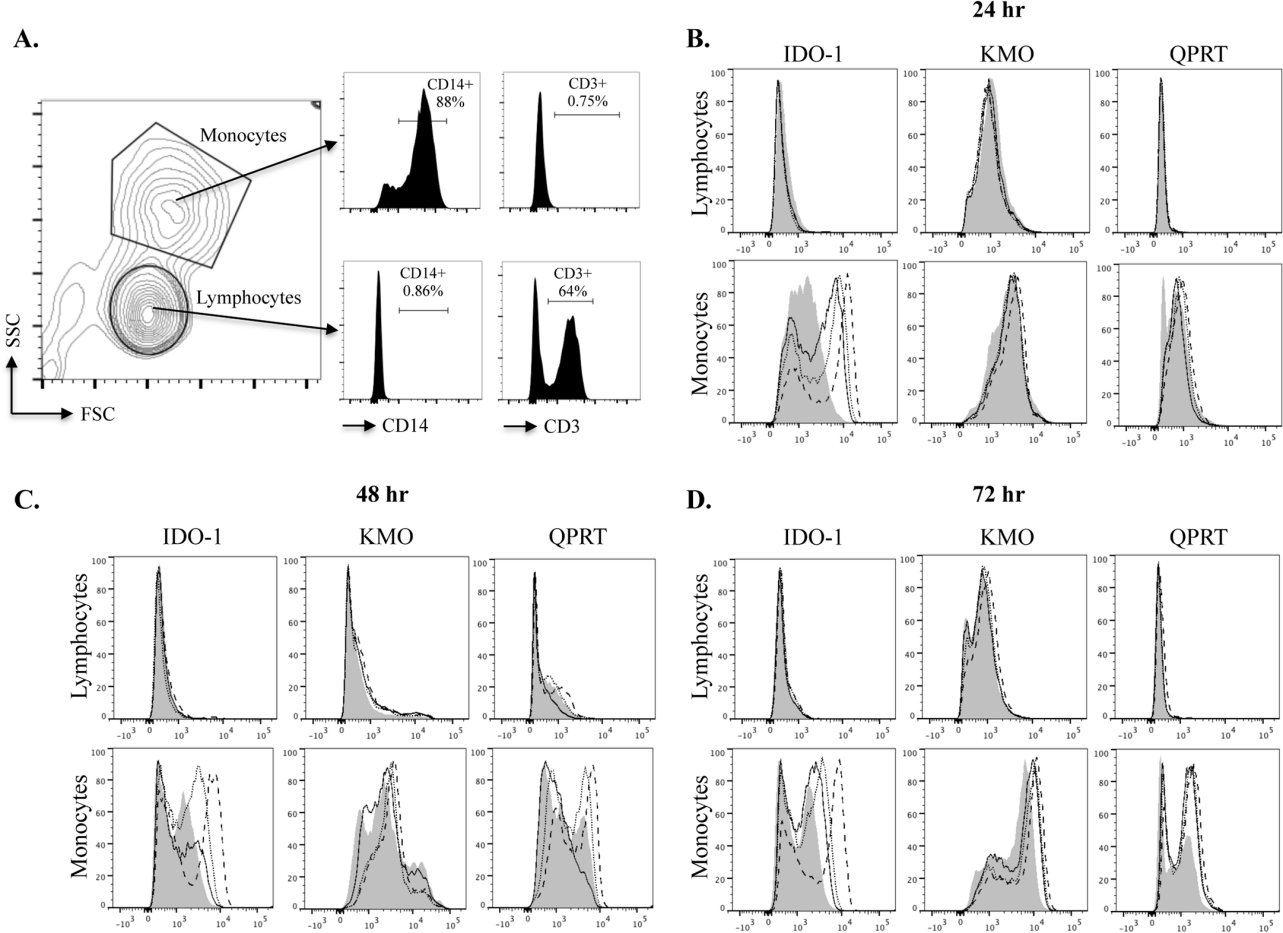
Expression of IDO-1, KMO and QPRT in lymphocytes and monocytes treated with IFN-γ. Flow cytometry histogram plots are based on forward scatter (FSC) and side scatter (SSC) gating for lymphocyte and monocyte populations. This was validated using CD14 and CD3 staining in some experiments. A representative example of the gating strategy is shown in (**A**). For histogram plots the mean fluorescence intensity (MFI) of expression for IDO-1, KMO and QPRT is represented on the *x* axis and normalized event numbers on the *y* axis at 24 hours (**B**), 48 hours (**C**) and 72 hours (**D**) of IFN-γ treatment. Untreated (control) cells are shown as the filled distribution, 50 IU/mL IFN-γ solid lines, 100 IU/mL IFN-γ dotted lines and 500 IU/mL IFN-γ dashed lines. Data is representative of at least 5 independent experiments.

Following fixation, PBMCs were washed in buffer containing 0.1% Tween20 (Sigma-Aldrich) to permeabilize the cell membrane and stained for 30 minutes in the dark at 4°C with Alexa Fluor 488 conjugated anti-human IDO-1, anti-human KMO or anti-human QPRT and the corresponding isotype control and secondary antibodies (Table 1). Gating for lymphocyte and monocyte populations was based on forward scatter and side scatter profiles. This was validated by surface staining cells with anti-CD3 and anti-CD14 antibodies in some experiments (Fig 2A). Following gating on these populations IDO-1, KMO and QPRT expression was visualized with histogram plots using FlowJo software (vX.0.6 Treestar). Geometric mean fluorescence intensity (gMFI) was computed for untreated lymphocytes/monocytes and IFN-γ (500 IU/mL) treated lymphocytes/monocytes. These values were corrected, in each independent experiment, for background fluorescence by subtracting the gMFI of cells treated with isotype control or secondary antibodies alone from the untreated and treated expression values.

**Table 1:** Antibodies used in flow cytometry experiments.

Target	Species	Isotype	Clone	Source	Fluorochrome
Human IDO	Mouse	IgG1	700838	R&D Systems	Alexa Fluor 488
Human KMO	Rabbit	Polyclonal	n/a	LSBio	Unconjugated
Human QPRT	Mouse	IgG1	7A8	LSBio	Unconjugated
Isotype control (MOPC-21 Ig)	Mouse	IgG1	MOPC-21	Becton Dickenson	Alexa Fluor 488
Mouse IgG1	Rat	IgG1	A85-1	Becton Dickenson	APC
Rabbit IgG	Goat	Polyclonal	n/a	Becton Dickenson	FITC
Human CD3	Mouse	IgG1	UCHT1	Biolegend	PerCP/Cy5.5
Human CD14	Mouse	IgG2a	M5E2	Becton Dickenson	Pacific Blue

IDO-1: indoleamine 2,3-dioxygenase; KMO: kynurenine 3-monoxygenase; QPRT: quinolinate phosphoribosyltransferase; Ig: immunoglobulin; APC: allophycocyanin; FITC: fluorescein isothiocyanate.

### Ultra High Performance Liquid Chromatography (UHPLC) and Gas Chromatography-Mass Spectrometry (GC-MS)

Supernatant samples were filtered with 0.22μm syringe filters before injecting into analysers. Concurrent quantification of TRP, KYN, 3-HK, 3HAA, AA and neopterin was carried out as previously described by our group [48] with the addition of 3HK, 3HAA, AA and neopterin analysed using the same method. An Agilent 1290 infinity ultra-high performance liquid chromatography system coupled with temperature controlled auto-sampler and column compartment, diode array detector and fluorescence detector was used for the analysis of these metabolites with a 20μL sample injection volume. Separation of metabolites was performed under stable temperature of 38°C for 12min, using 0.2mM sodium acetate (pH 4.65) as mobile phase, with an isocratic flow rate of 0.75ml/min in an Agilent Eclipse Plus C18 reverse-phase column (2.1mm x 150mm, 1.8μm particle size). 3HK and KYN were detected using UV wavelength at 365 nm with a retention time of 1.2 and 3.1 min, respectively. TRP, 3HAA and AA were detected using fluorescence intensity set at Ex/Em wavelength of 250/438 for neopterin, 280/438 for TRP and 320/438 for 3HAA and AA. The retention times for neopterin, TRP, 3HAA and AA are 1, 7.4, 3.3 and 9.8 min, respectively. Mixed standards of all metabolites were used for a six-point calibration curve in order to interpolate the quantity of the sample readout. Agilent OpenLAB CDS Chemstation (Edition C.01.04) was used to analyze the chromatogram. The inter- and intra-assay coefficient of variation is within the acceptable range of 3–7%. PIC and QUIN were detected using GC-MS in accordance to our method previously described in [49]. Representative UPLC and GC-MS profiles for the kynurenine metabolites are provided in Fig D in S1 File.

### Statistical analysis

All data are presented graphically as raw values unless stated otherwise. Where appropriate these data have been log^10^ transformed to obtain a normal distribution for statistical analysis. For analysis of differences between time-points, gMFI values for IDO-1, KMO and QPRT at 24, 48 and 72 hours were compared with the Tukey test. For pairwise comparison of differences between control and treatment groups a Dunnett’s test was used. Correlations were performed on log^10^ transformed data using bivariate *x* and *y* fit modelling. All statistical analysis was performed using JMP v10 SAS software for Macintosh.

## Results

### KP up regulation does not occur in lymphocytes

**Fig 3:**
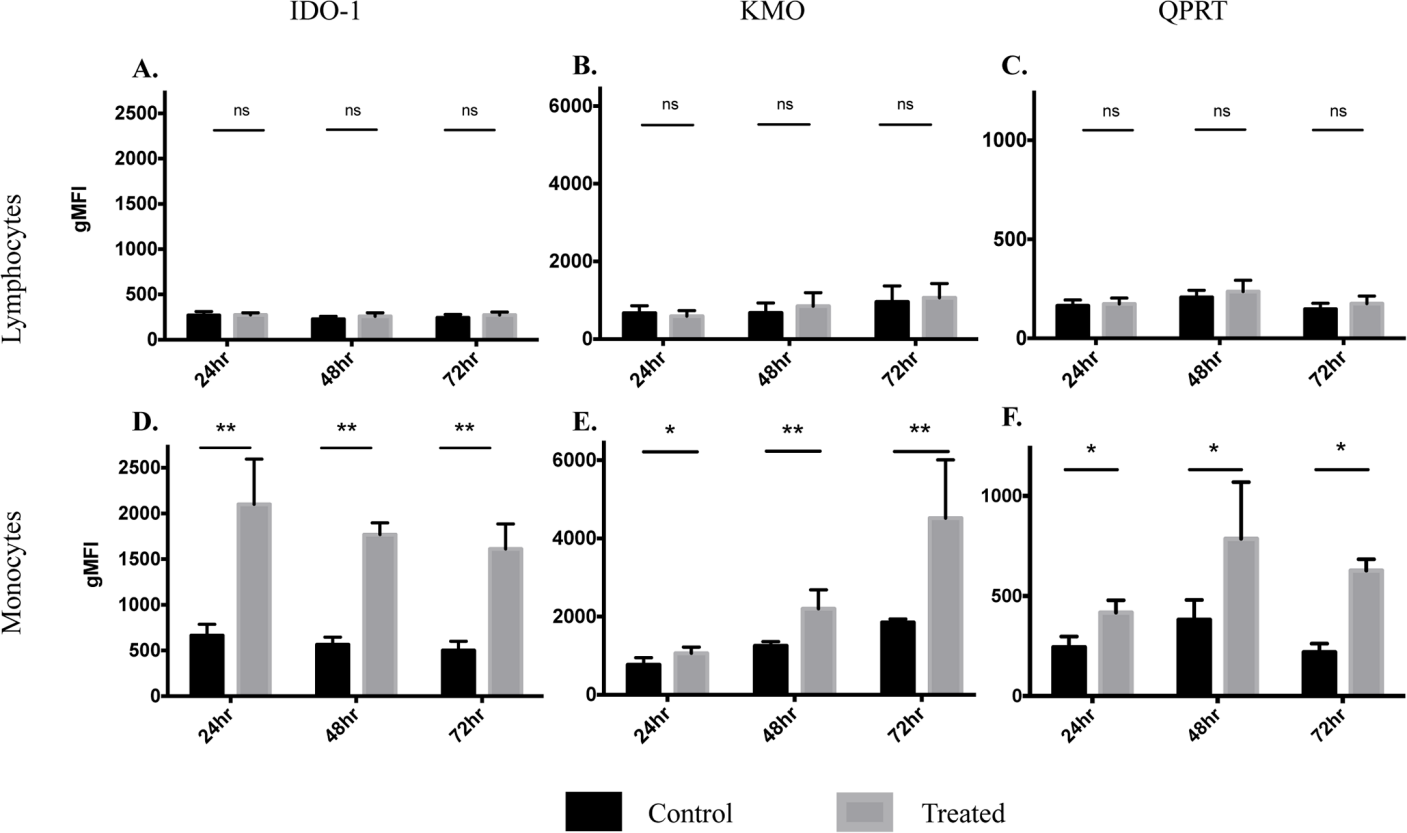
Geometric mean fluorescence intensity (gMFI) for IDO-1, KMO and QPRT expression. A summary of gMFI expression for the three KP enzymes in lymphocytes (**A-C**) and monocytes (**D-F**). Data is from 5 different biological samples with time-point (24, 48 and 72 hours) shown on the *x* axis. Black bars represent control experiments (untreated) and grey bars represent treated (500 IU/mL IFN-γ). The *y* axis scale for gMFI has been normalized to improve comparison of expression for IDO-1, KMO and QPRT between lymphocytes (**upper row**) and monocytes (**lower row**). Data is presented as mean±SEM, ns = no significant difference, *p<0.01, **p<0.001.

We validated our forward scatter and side scatter gating strategy using CD14 and CD3 staining in some experiments (Fig 2A). The monocyte gate was consistently 80–85% CD14+ and negative for CD3. The lymphocyte gate 60–70% CD3+ and negative for CD14 (Fig 2A). Furthermore, we saw no significant differences in the expression of KP enzymes when comparing lymphocyte gate to CD3+ cells and when comparing our monocyte gate to CD14+ cells (data not shown). We consistently observed no significant increase in the expression of IDO-1, KMO or QPRT in lymphocyte populations stimulated with IFN-γ at all doses tested. This is shown in the representative flow cytometry histograms for the lymphocyte gate in Fig 2B–2D, as well as the graphs depicting the lymphocyte gate gMFI values for 5 independent biological samples (Fig 3).

There was no significant effect of time in culture on levels of IDO-1 (p = 0.6993), KMO (p = 0.6803) or QPRT (p = 0.2181) in lymphocytes. We also observed no significant overall treatment effect, independent of time-point, between control and treated (500 IU/mL IFN-γ) samples: IDO-1 p = 0.3638, KMO p = 0.8337 and QPRT p = 0.5091 (Fig A in S1 File).

### KP enzymes are significantly up regulated in monocytes

We found a dose-dependent increase in the expression of IDO-1, KMO and QPRT in monocyte populations stimulated with IFN-γ (Fig 2B–2D). At doses above 500 IU/mL IFN-γ we observed no further significant increases in KP enzyme expression (data not shown). Therefore we chose 500 IU/mL as the optimal dose for KP activation and this is represented as ‘treated’ cells in Fig 3. IDO-1 expression was significantly elevated in IFN-γ treated monocytes at 24 hours with elevated expression continuing at 48 and 72 hours (Fig 3D). KMO expression was modestly but significantly elevated with IFN-γ treatment at 24 hours then continued to progressively increase at 48 hours, with the most significant increase at 72 hours (Fig 3E). At all three time-points the expression of QPRT was less markedly up regulated with IFN-γ treatment than for IDO-1 and KMO. However, differences between control and treated monocytes were still significant and became progressively more so over time, with QPRT elevation peaking at 72 hours (Fig 3F).

Overall there was no significant effect of time in culture on levels of IDO-1 (p = 0.3187) and QPRT (p = 0.1009) in monocytes. However, time did exert a significant effect, independent of treatment, on KMO expression across the three time-points (p<0.0001) (Fig B in S1 File). Importantly we observed highly significant treatment effects, independent of time-point, between control and treated (500 IU/mL IFN-γ) samples for all three KP enzymes: IDO-1 p<0.0001, KMO p = 0.0023 and QPRT p = 0.0002 (Fig B in S1 File).

For a full description of the statistical differences in IDO-1, KMO and QPRT expression between control and treated samples for both lymphocyte and monocyte populations at each time-point, see Table 2.

**Table 2:** Significance difference levels between control and treatment groups for IDO-1, KMO and QPRT expression (gMFI) for both lymphocytes and monocytes at each time-point tested.

Cell type	KP enzyme	Time-point (hours)	Control vs treated (*p* value)
**Lymphocytes**	IDO-1	24	ns 0.9988
	IDO-1	48	ns 0.9503
	IDO-1	72	ns 0.9332
	KMO	24	ns 1.0000
	KMO	48	ns 0.9986
	KMO	72	ns 0.9979
	QPRT	24	ns 0.9997
	QPRT	48	ns 0.9850
	QPRT	72	ns 0.9791
**Monocytes**	IDO-1	24	** 0.0006
	IDO-1	48	** 0.0003
	IDO-1	72	** 0.0003
	KMO	24	* 0.0015
	KMO	48	** 0.0008
	KMO	72	** 0.0006
	QPRT	24	* 0.0078
	QPRT	48	* 0.0047
	QPRT	72	* 0.0038

Data was log10 transformed to normalise distributions and pairwise differences between control and treatment groups analysed using a Dunnett’s test (*p<0.01, **p<0.001, ns: not significant). gMFI: geometric mean fluorescence intensity; KP: kynurenine pathway; IDO-1: indoleamine 2,3-dioxygenase; KMO: kynurenine 3-monoxygenase; QPRT: quinolinate phosphoribosyltransferase.

### Production of KP metabolites validates KP enzyme expression

**Fig 4:**
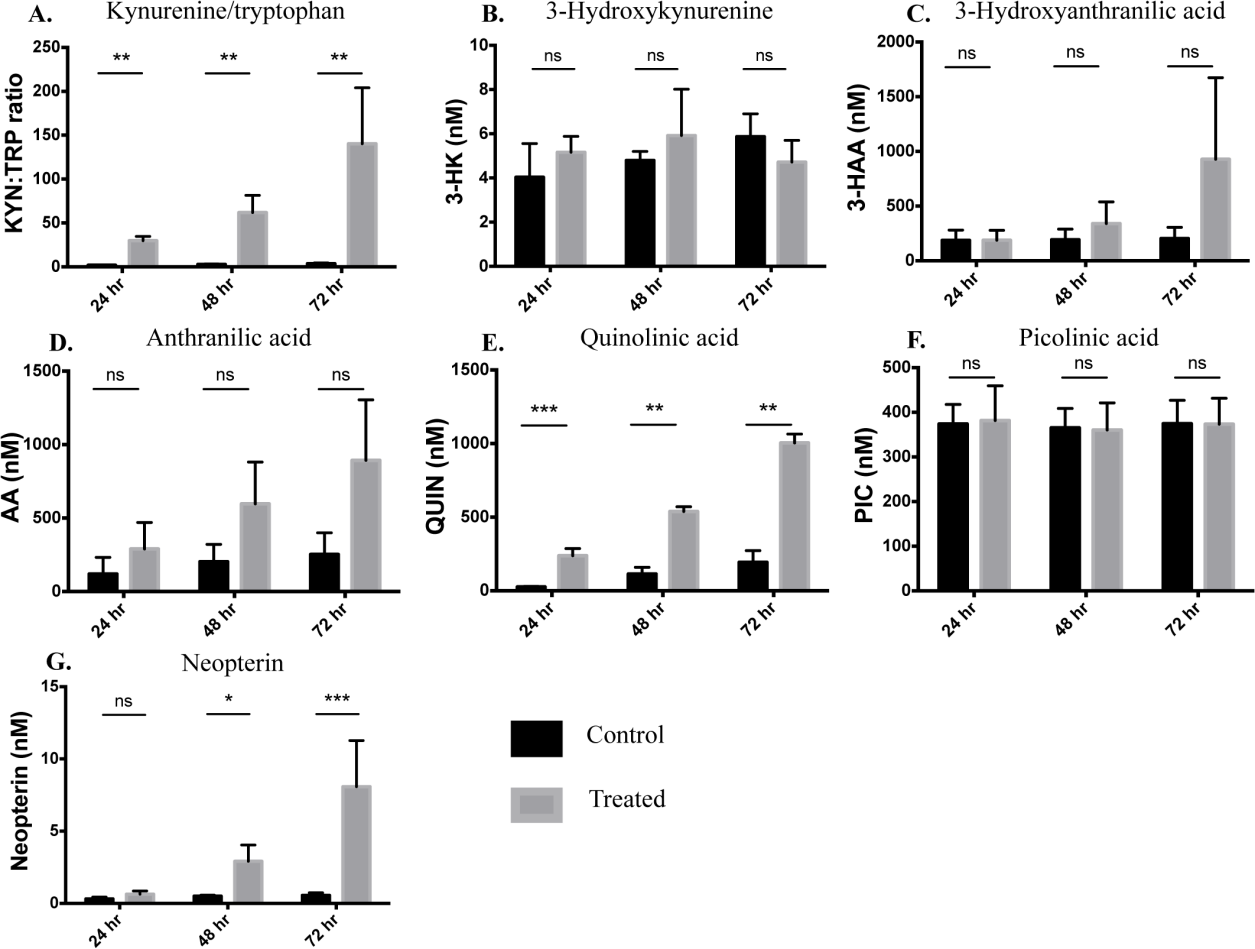
Concentrations of KP metabolites in the supernatants of IFN-γ activated PBMCs. Levels of KP metabolites: tryptophan (TRP) and kynurenine (KYN)-shown as TRP:KYN ratio (**A**); 3-hydroxykynurenine (3-HK) (**B**); 3-hydroxyanthranillic acid (3-HAA) (**C**); anthranilic acid (AA) (**D**); quinolinic acid (QUIN) (**E**); picolinic acid (PIC) (**F**) and the pro-inflammatory marker: neopterin (**G**) were measured by UHPLC or GC-MS (QUIN and PIC only) in the supernatants of PBMCs untreated (control-black bars) or treated with IFN-γ (500 IU/mL-grey bars) at 24, 48 and 72 hours of culture. Values represent the mean±SEM of 4 independent biological repeats (ns = no significant difference, *p<0.05, **p<0.01, **p<0.001).

We assessed the levels of major KP metabolites in the supernatant of PBMC either untreated (control) or treated with 500 IU/mL IFN-γ. The ratio of kynurenine to tryptophan (KYN:TRP, a sensitive measure of IDO-1 activation) was significantly increased at all time-points (p<0.0001). This was largely due to an increase in KYN production not a decrease in TRP, which remained relatively constant (Fig C in S1 File). The highest KYN:TRP ratio was seen at 72 hours (Fig 4A). Concentrations of 3-HK and 3-HAA were not significantly elevated in cultures of IFN-γ treated PBMC compared to control cultures at any time-point (Fig 4B & 4C). However, whilst not statistically significant, there was a clear trend for 3-HAA to be elevated in treated cultures at 72 hours (Fig 4C). Similarly, there was a strong trend for AA levels to be elevated in IFN-γ treated PBMC cultures with progressive increase over time, although this did not reach statistical significance (Fig 4D).

Concentrations of QUIN were highly significantly elevated in cultures of IFN-γ treated PBMC compared to control cultures, at all time-points (Fig 4E). The difference between control and treated PBMC was most statistically significant at 24 hours. However, absolute levels of QUIN increased over time and peaked at 72 hours. We saw no significant differences in levels of PIC between control and treatment groups at all time-points (Fig 4F). Concentrations of neopterin—a useful marker of a Th1 type immune response, derived from activated monocytic cells—were significantly elevated in treated PBMC at 48 and 72 hours with the highest levels observed at 72 hours (Fig 4G).

**Fig 5:**
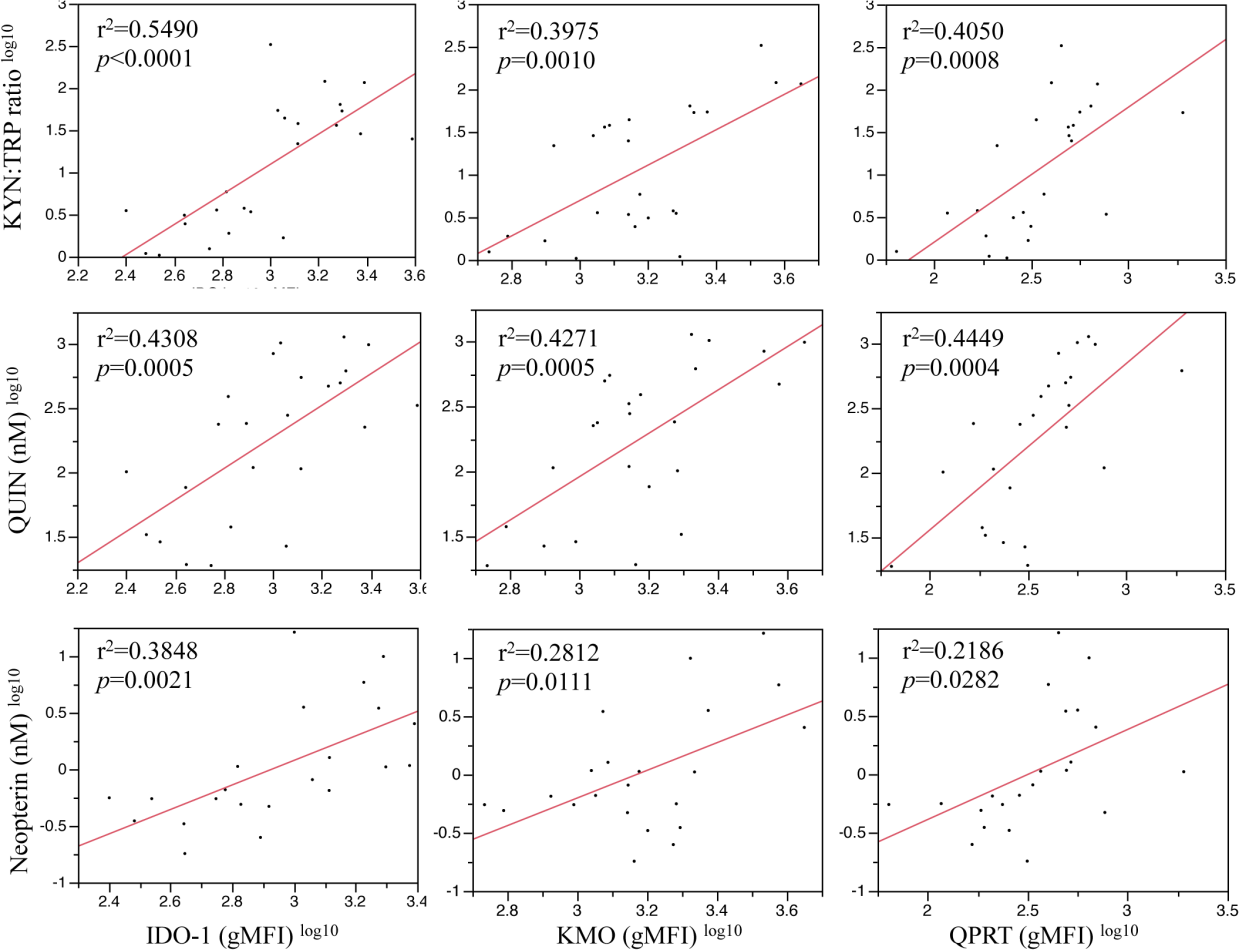
Correlations of IDO-1, KMO and QPRT expression (gMFI) with KYN:TRP ratio, QUIN concentration (nM) and neopterin levels (nM). Data was log^10^ transformed to normalize distributions. Straight-line fits show least squares regression with the r^2^ value providing a summary of fit. *P* values were computed using ANOVA for a regression analysis of the total variation for each correlation (n = 24).

We found significant correlations between the expression of IDO-1, KMO and QPRT with KYN:TRP ratio, QUIN concentration and neopterin levels (Fig 5). We also observed a significant correlation between 3-HK and QPRT (r^2^ = 0.2746, *p* = 0.0086, data not shown).

## Discussion

We have shown for the first time that human peripheral blood monocytes significantly up-regulate three major enzymes of the KP (IDO-1, KMO and QPRT) in a *time dependent* manner, when treated with IFN-γ. Importantly, we also describe for the first time that lymphocytes do not express the three KP enzymes investigated, even under stimulation with IFN-γ (Fig 2B–2D and Fig 3A–3C). From a novel methodological perspective, flow cytometry was successfully used to quantify the intracellular expression of IDO-1, KMO and QPRT in human PBMCs (Fig 2). All three enzymes were significantly increased in monocytes following IFN-γ treatment. However, this occurred to different extents and in varying temporal dynamics between the three enzymes: IDO-1 increased robustly by 24 hours and maintained a significant elevation at 48 and 72 hours (Fig 3D). KMO showed a slower and progressive increase in expression, peaking at 72 hours (Fig 3E). QPRT displayed much lower levels of expression and was less significantly up regulated overall, peaking at 48–72 hours (Fig 3F **)**.

Our gating strategy to distinguish monocytes and lymphocytes is based on forward and side scatter parameters, validated by staining for CD14 and CD3 in some experiments (Fig 2A). A valid criticism is that the monocyte gate might be better termed the ‘non-lymphocyte’ gate, as it contains some cells (typically 15–20%) that are CD14^-^, with virtually all cells CD3^-^. We observed no significant difference in the expression of IDO-1, KMO and QPRT in CD14^+^ cells compared with our monocyte gate and similarly in CD3^+^ cells compared with our lymphocyte gate (data not shown). This excludes the possibility that CD14^-^ cells are responsible for the elevated KP enzyme levels seen in the monocyte gate.

The KP metabolite concentrations in the supernatant of IFN-γ stimulated PBMCs, quantified using UHPLC and GC-MS, fully supported our flow cytometry data. KYN/TRP ratio, a sensitive measure of IDO-1 activation, became progressively elevated over the time-course, peaking at 72 hours (Fig 4A). This was due largely to increased production of KYN rather than reduced TRP (Fig C in S1 File). This diminishes the likelihood of possible differences to the *in vivo* situation due to TRP depletion in media, as our data suggest this was not a limiting factor. We have previously observed that in patients, TRP depletion is unlikely to be a significant phenomenon [50]. We found no significant elevation in 3-HK concentrations (Fig 4B). 3-HAA and AA levels showed a clear trend to progressively increase with time, however this did not reach statistical significance (Fig 4C & 4D). Concentrations of the QUIN reached excitotoxic levels (150–500 nM range) by 24 hours and this continued to rise, attaining micromolar concentrations by 72 hours (Fig 4E). Interestingly, levels of neuroprotective PIC remained unchanged between control and treated PBMC cultures at all time-points assessed (Fig 4F). Importantly we show that the monocyte-derived marker of a Th1 type immune response, neopterin, was significantly elevated in cultures of treated PBMCs by 48 hours, and this further increase at 72 hours (Fig 4G). To further validate our flow cytometry results, analysis of the relationships between IDO-1/KMO/QPRT and KP metabolite expression revealed that KYN:TRP ratio, production of QUIN and levels of neopterin were strongly correlated with expression of all three KP enzymes (Fig 5).

Our finding that KP is significantly up regulated in peripheral blood monocytes confirms previous findings [43–45, 47]. The dynamics and temporal expression of KP enzymes following activation and their relationship to KP metabolite production have not been previously reported. Up regulation of IDO-1 in monocytes is part of a highly conserved, endogenous regulatory mechanism to suppress immune responses and protect local tissues. The immunological suppression resulting from IDO-1 up regulation is well documented and results from at least three mechanisms that are not thought to be mutually exclusive. Firstly, the depletion of the essential amino acid TRP by increased IDO-1 activity has an anti-proliferative [51] and pro-apoptotic effect [52] on T cells. Secondly intermediate KP metabolites, such as 3-HK and 3-HAA, are known to suppress the proliferation of activated Th1 cells [18, 53, 54]. Thirdly, activation of the KP induces tolerogenic dendritic cells [55, 56] as well as T cells with a regulatory phenotype and down regulates the CD3ζ chain on activated T cells, at least in mice [57]. More recently, the highly evolutionary conserved Aryl hydrocarbon receptor (AhR) has also received attention for its mechanistic role in linking tryptophan metabolism with immune regulation [58]. Experiments in mice have revealed that KYN selectively activates the AhR and induces the generation of FoxP3^+^ T regulatory cells [59]. Our data suggests that peripheral monocytes are crucial in mediating at least some of these immunosuppressive effects and this warrants further investigation. The inherent lack of KP activation in lymphocytes implies they serve as effector cells responding to changes in the cellular environment resulting from KP activation in monocytes. This has important implications for therapies aimed at the KP, especially in terms of selectively targeting monocytic cells. Last year White *et al* showed that a microparticle, known as MIS416 (originally devised as an adjuvant in vaccines) was effective at attenuating the autoimmune response in experimental autoimmune encephalomyelitis (EAE) [60]. MIS416 is now in phase 2a clinical trials for the treatment of patients with secondary progressive multiple sclerosis (SPMS). MIS416 specifically targets myeloid cells by activating innate pattern recognition receptors TLR9 and NOD2. Administration of MIS416 increased IFN-γ in mice with EAE and elevated IFN-γ was also observed in SPMS patients [60]. These findings raise the intriguing possibility that MIS416 may exert its disease modifying effects through activation of the KP in monocytes. The importance of focusing treatment strategies for neuroinflammatory diseases on peripheral monocytes was further highlighted with the recent discovery that peripheral blood monocyte derived macrophages, not brain resident microglia, mediated axon destruction and demyelination [61].

We observed a rapid initial increase in the expression of IDO-1 by 24 hours and at 72 hours levels of IDO-1 were still significantly elevated. Additionally, we have preliminary observations that IDO-1 expression remains elevated at 7 days following treatment with IFN-γ (unpublished data). The early induction of IDO-1 in response to pro-inflammatory insult is evidently important for regulating T cell responses, as discussed above. However, there is a synergistic effect of QUIN and pro-inflammatory molecules, such as IL-1β [62] and 3-HK [63], exacerbating KP activation. Furthermore, the latter has been shown to be pro-excitotoxic and potentiates neurodegenerative disease [64]. Thus, if monocytes remain exposed to an inflammatory milieu, IDO-1 and KMO expression is chronically elevated. This results in the increasing accumulation of free-radical generator 3-HAA, AA and neurotoxic/gliotoxic QUIN (Fig 4D–4F), leading to chronic inflammatory and neurodegenerative effects [65]. We have results from human MS patients that QUIN is elevated to neurotoxic and gliotoxic levels in those with progressive disease (unpublished data). There is also an expanding body of evidence that links immunoregulation and altered pro-inflammatory cytokine levels with dysregulated KP metabolism in the context of neurodegenerative and psychiatric diseases (for a review, see [42]). Our finding that levels of neopterin increase progressively over time, further supports the role of monocytes in these pathogeneses (Fig 4G).

Apart from IDO-1, the regulation of other KP enzymes by pro-inflammatory cytokines has not been well studied. Published findings from our laboratory showed that human mesenchymal stem cells increase KMO expression at the RNA level following IFN-γ and IFN-β stimulation [66]. The only other data from human cells demonstrated that treatment with IL-1β up-regulated transcripts for KMO in hippocampal progenitor cells [67]. To our knowledge there is currently no evidence that IFN-γ directly induces KMO. Any increase in KMO expression is likely a response to increased levels of its substrate, KYN, or other inflammatory mediators such as LPS, TNF-α or IL-6 [68]. We demonstrate for the first time that KMO is significantly elevated after 24 hours of stimulation with IFN-γ in human peripheral monocytes and this increases at 48 and 72 hours. Statistical modelling revealed that KMO expression also increased over time, independent of IFN-γ treatment (Fig B in S1 File). This is further evident in the untreated control conditions in Fig 3E. Increased basal KMO expression over time likely results from the increased KYN present in the PBMC supernatant (Fig C in S1 File) and supports the hypothesis that IFN-γ does not directly induce KMO.

To validate our flow cytometry data for KMO expression, we show that KMO downstream metabolites: 3-HAA, AA and QUIN increase in the supernatant of IFN-γ treated PBMC cultures (Fig 4D–4F). KMO acts as the ‘gatekeeper’ to the largely neurotoxic arm of the KP, which results in production of neurotoxic 3-HK, the ROS generating 3-HAA and the terminal metabolites QUIN or PIC. Our finding that 3-HK is not significantly up regulated in PBMC cultures with IFN-γ treatment (Fig 4B) is not surprising as 3-HK is rapidly catabolised to the next metabolite(s). 3-HK can undergo transamination to form xanthurenic acid or is catalysed by kynureninase along another branch to form 3-HAA. Furthermore, 3-HK easily oxidizes and has also been shown to act as an anti-oxidant in the cellular environment by rapidly scavenging reactive oxygen species [69]. We observed that both AA and 3-HAA showed a clear trend to up regulate over the study time-course. However, there was considerable variation in AA and 3-HAA levels between PBMC donors and the effect of IFN-γ treatment did not reach significance. 3-HAA can be converted from both 3-HK and AA; in the EAE model of multiple sclerosis, 3-HAA produced as a result of KP activation has been shown to enhance the production of TGF-β in dendritic cells, which in turn promoted regulatory T cell differentiation [70]. Interestingly, using the same animal model, a synthetic homologue of AA (3,4,-Dimethoxycinnamoyl anthranilic acid or 3,4-DAA) has been shown to be effective at inhibiting the proliferation of auto-reactive T cells, reducing pro-inflammatory cytokine production and alleviating disability in EAE mice [71]. The authors suggest that suppression of monocytic antigen presenting cells is a key mechanism by which 3,4-DAA exerts its immunosuppressive effect. Our findings support the pivotal role of peripheral monocytes in this process.

A limitation of the currently study is that we do not have the complete picture indicating levels of neuroactive kynurenic acid (KYNA) in our experimental model. KYNA is the end product of an alternative branch of the KP whereby KYN is catabolized by the kynurenine aminotransferase (KAT) family of enzymes (Fig 1). KYNA is an NMDA receptor agonist [72] and elevation of KYNA has been found to provide neuroprotection in a number of neurological pathologies, including: Alzheimer’s disease, Parkinson’s disease, Huntington’s disease, amyotrophic lateral sclerosis, migraine and epilepsy [42]. Our preliminary results indicate that KYNA levels in the supernatant of PBMCs increases in a dose and time dependent manner with IFN-γ treatment (data not shown). Interestingly KYNA has been shown to bind the previously orphaned G protein-coupled receptor GPR35 on monocytes and inhibit their LPS induced TNF-α secretion [73]. The expression of GPR35 on CD14^+^ cells and the observed increase in KYNA with inflammatory insult suggest that this receptor-ligand interaction plays an important role in immune regulation and should be the focus of further investigation. It also highlights the need to focus therapeutic interventions on promoting the production of KYNA (neuroprotective arm) and inhibiting the neurotoxic arm, which culminates in the production of QUIN. Chemical inhibitors of KMO represent one such strategy that might address this need.

The ‘neurotoxic arm’ of the KP can also result in the production of PIC, which is an endogenous neuroprotectant and metal chelator but can have toxic effects at high concentrations [74]. PIC also has an immunomodulatory role and has been shown to selectively suppress proliferation of activated T cells [18]. We observed no increase in PIC concentrations beyond those normally seen in human serum (100–400 nM) [75]. Like KYNA, PIC can antagonise the neurotoxic effects of QUIN, however, our data suggests this is unlikely to be the case with IFN-γ activated PBMCs. This lends further credence to the hypothesis that continued stimulation of the KP in monocytes promotes a dysregulated inflammatory environment with the preferable accumulation of cytotoxic mediators like QUIN.

A confounding effect on the increased levels of QUIN described here, is our finding that QPRT expression was relatively low compared to both IDO-1 and KMO (Fig 3F) and showed the least significant up regulation with IFN-γ treatment (Table 2). Our group and others have shown previously that QUIN accumulates in monocytic cells following inflammatory stimulus [7, 76]. This leads to the possibility that QPRT becomes saturated upon long-term exposure to inflammation and this exacerbates the high levels of QUIN. In fact, we have previously shown that in human primary neurons QPRT activity becomes saturated in the presence of high extracellular concentrations of QUIN (>300 nM) [77]. The concentrations of QUIN observed in this study far exceeded 300 nM by 48 hours IFN-γ stimulation (Fig 4E). Elevated levels of QPRT in glioma have been shown to be cytoprotective and thus, in that situation, confer poorer prognosis as QPRT increases the tumours resistance to radiochemotherapy [78]. In the context of neuroinflammatory and degenerative diseases, where elevated QUIN contributes to the pathogenesis, increasing expression or activity of QPRT may provide a useful therapeutic strategy.

We have characterised for the first time the temporal dynamics of KP activation by quantifying, using a novel approach, the expression of IDO-1, KMO and QPRT in human PBMCs. Importantly we demonstrate the relationship these changes in KP enzyme expression have at the metabolite level. This provides important insights into the role of KP activation in inflammatory and neurodegenerative disease pathogenesis. It also highlights potential therapeutic targets, some of which are already under investigation. In future work we will better characterize the monocyte subpopulations important in mediating the immunomodulatory effects of KP activation. We also plan to address the important effects of a long-term, chronic inflammatory environment on KP expression and function in immune cells.

## Supporting Information

S1 File The effects of time in culture and treatment on IDO-1, KMO and QPRT expression in lymphocytes (Fig A). The effects of time in culture and treatment on IDO-1, KMO and QPRT expression in monocytes (**Fig B**). Concentrations of tryptophan (TRP) and kynurenine (KYN) in the supernatants of IFN-γ activated PBMCs (**Fig C**). UHPLC and GC-MS chromatogram profiles for KP metabolites and neopterin (**Fig D**).
(PDF)
