# Supplementary material for: Expression of the Kynurenine Pathway in Human Peripheral Blood Mononuclear Cells: Implications for Inflammatory and Neurodegenerative Disease
Source: PLoS One. 2015 Jun 26;10(6):e0131389. doi: 10.1371/journal.pone.0131389 (PMC4482723; doi:10.1371/journal.pone.0131389)
Supplement: S1 File — The effects of time in culture and treatment on IDO-1, KMO and QPRT expression in monocytes (Fig B). Concentrations of tryptophan (TRP) and kynurenine (KYN) in the supernatants of IFN-γ activated PBMCs (Fig C). UHPLC and GC-MS chromatogram profiles for KP metabolites and neopterin (Fig D). (PDF) [file pone.0131389.s001.pdf]

## Supporting information (S1 File)

**Fig. A. The effects of time in culture and treatment on IDO-1, KMO and QPRT expression in lymphocytes.** The leverage plots show the influence of time in culture (left column) and the effect of 500 IU/mL IFN-γ treatment (right column) on KP enzyme expression, modeled independently of other factors. The dashed horizontal line depicts no effect (null hypothesis) whilst the solid line is the line of fit for the observed data. 95% confidence curves (dashed, curved lines) for the line of fit provide a visual indication of whether the test of interest is significant: if the confidence region between the curves contains the dashed horizontal line then the effect is not significant; if the curves cross the line, the effect is significant. For the lymphocyte population in our study, both time in culture and treatment had no significant effect on KP enzyme expression.

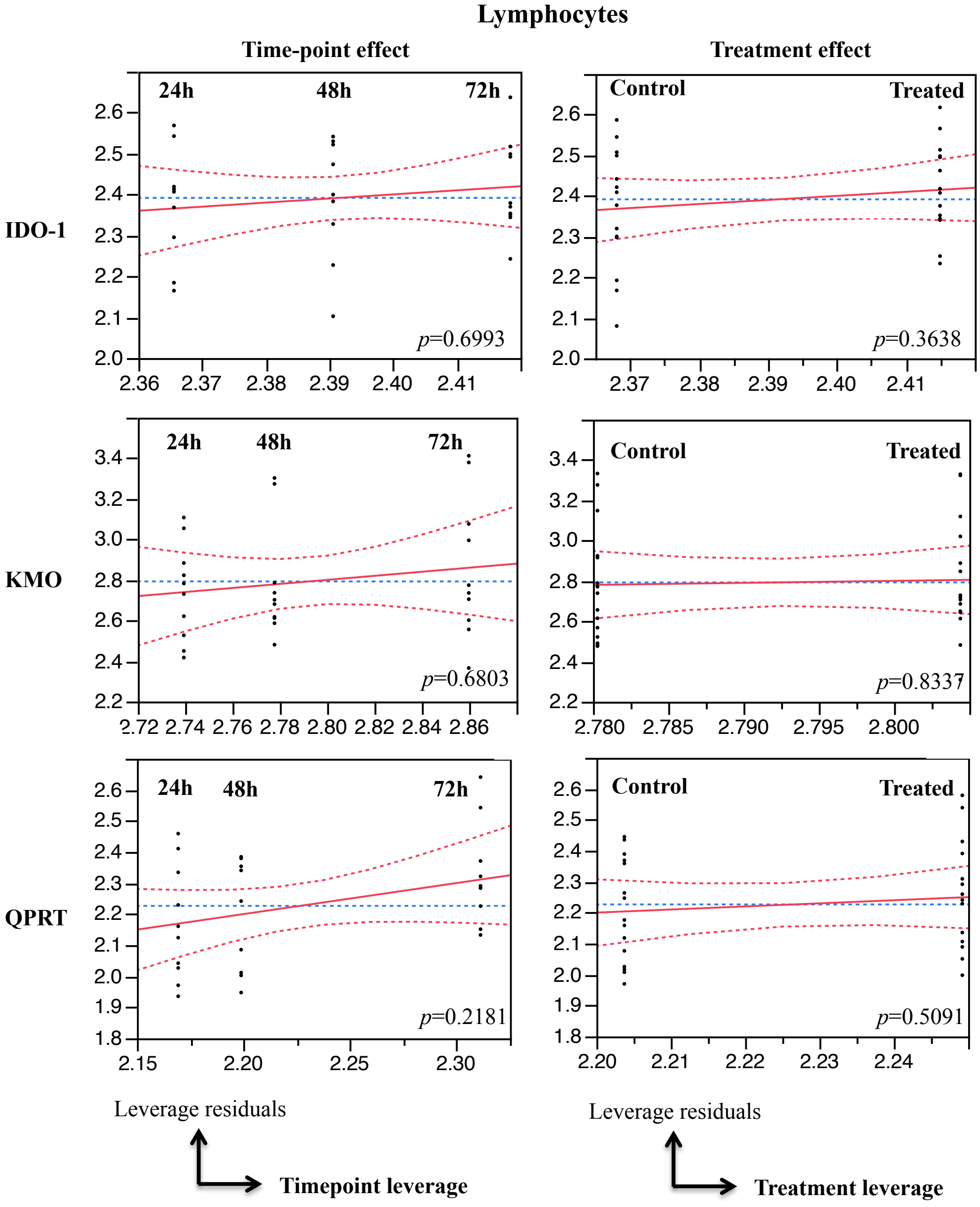

**Fig. B. The effects of time in culture and treatment on IDO-1, KMO and QPRT expression in monocytes.** The leverage plots show the influence of time in culture (left column) and the effect of 500 IU/mL IFN-γ treatment (right column) on KP enzyme expression, modeled independently of other factors. The dashed horizontal line depicts no effect (null hypothesis) whilst the solid line is the line of fit for the observed data. 95% confidence curves (dashed, curved lines) for the line of fit provide a visual indication of whether the test of interest is significant: if the confidence region between the curves contains the dashed horizontal line then the effect is not significant; if the curves cross the line, the effect is significant. For the monocyte population in our study, time in culture had no significant effect on IDO-1 and QPRT expression. However, time in culture alone did exert a highly significant effect on KMO expression in monocytes. Treatment with IFN-γ exerted a significant effect on expression of all 3 enzymes (right column).

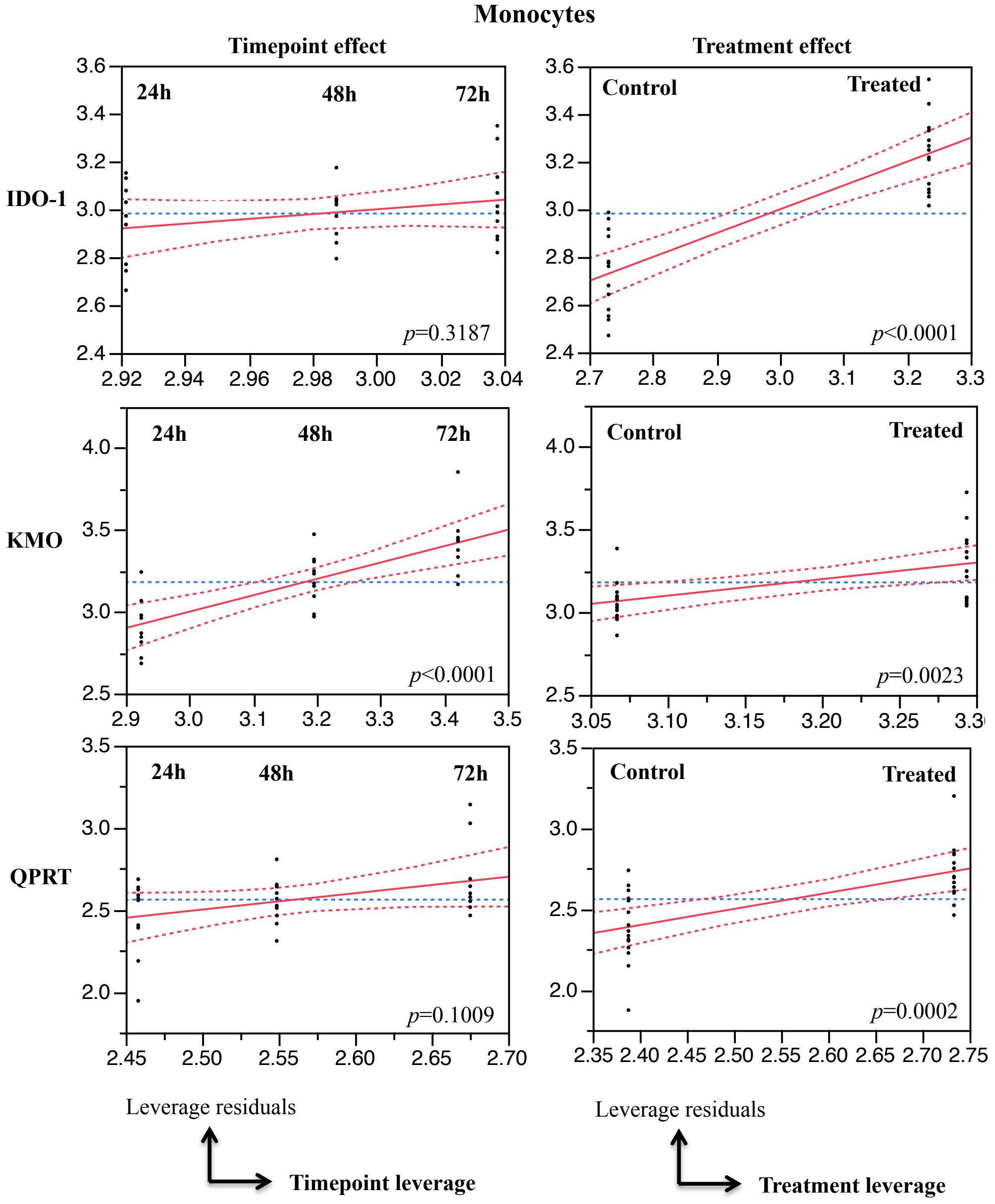

**Fig. C. Concentrations of tryptophan (TRP) and kynurenine (KYN) in the supernatants of IFN-γ activated PBMCs.** TRP and KYN were measured by UHPLC in the supernatants of PBMCs untreated (control – black bars) or treated with IFN-γ (500 IU/mL – grey bars) at 24, 48 and 72 hours of culture. Values represent the mean ± SEM of 4 independent biological repeats (ns = no significant difference, **p*<0.05, ***p*<0.01). TRP is not significantly reduced in treated PBMC cultures, therefore its depletion is unlikely to play a significant role in the experimental system. In contrast KYN is significantly up regulated providing increased substrate for downstream KP enzymes, such as KMO.

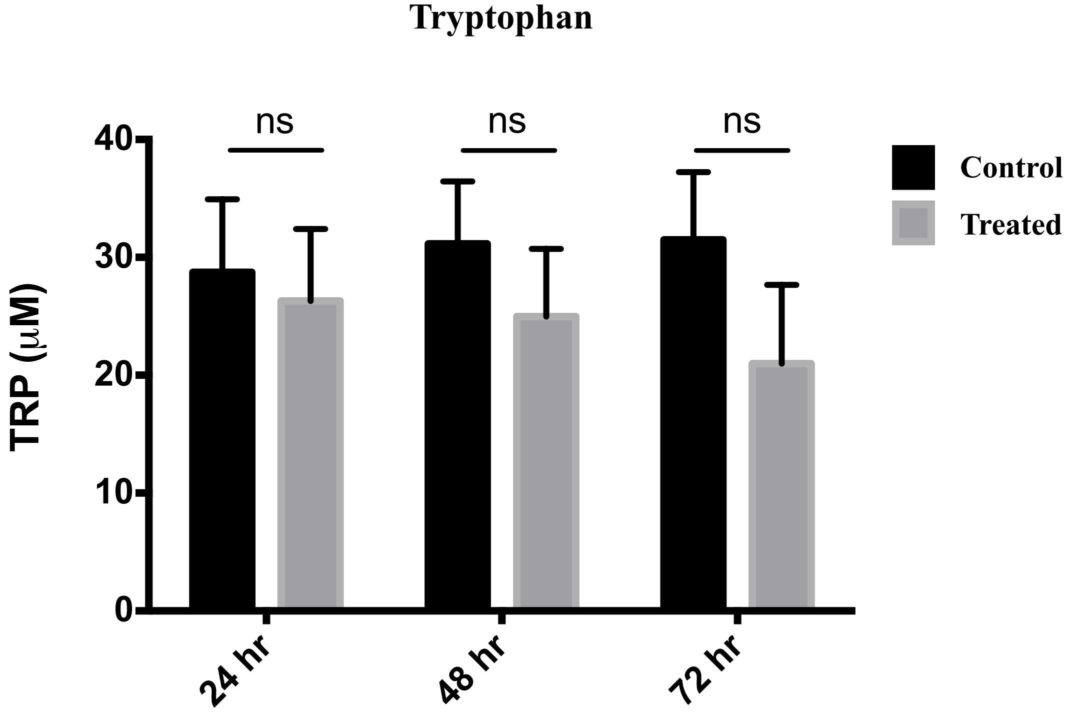


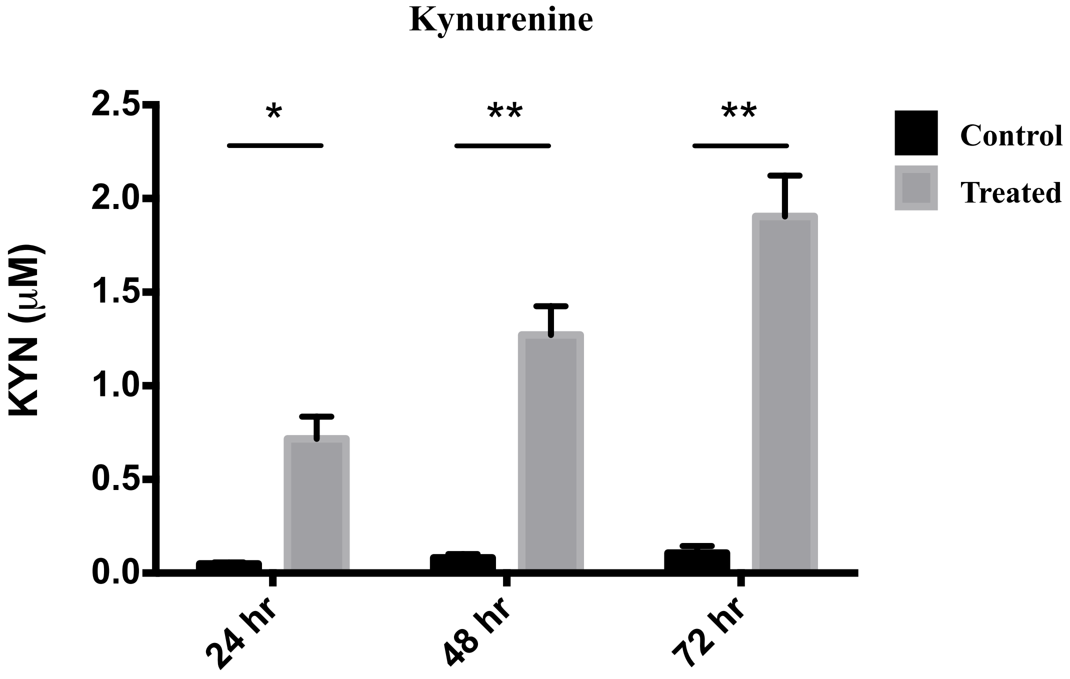

**Fig. D. UHPLC and GC-MS profiles for KP metabolites.** Chromatogram showing concurrent detection of 3-HK, 3-HAA, AA and Neopterin (**A**). Chromatogram showing concurrent detection of TRP and KYN (**B**). Representative sample showing profiles of all six metabolite peaks detected using UHPLC (**C**). **A-C** produced using UHPLC 1290 Agilent Openlab software. Chromatogram showing standard profiles for PIC and QUIN (**D**) and chromatogram of showing PIC and QUIN peaks in a representative sample (**E**) both using GC-MS Agilent Chemstation software.

**A.**

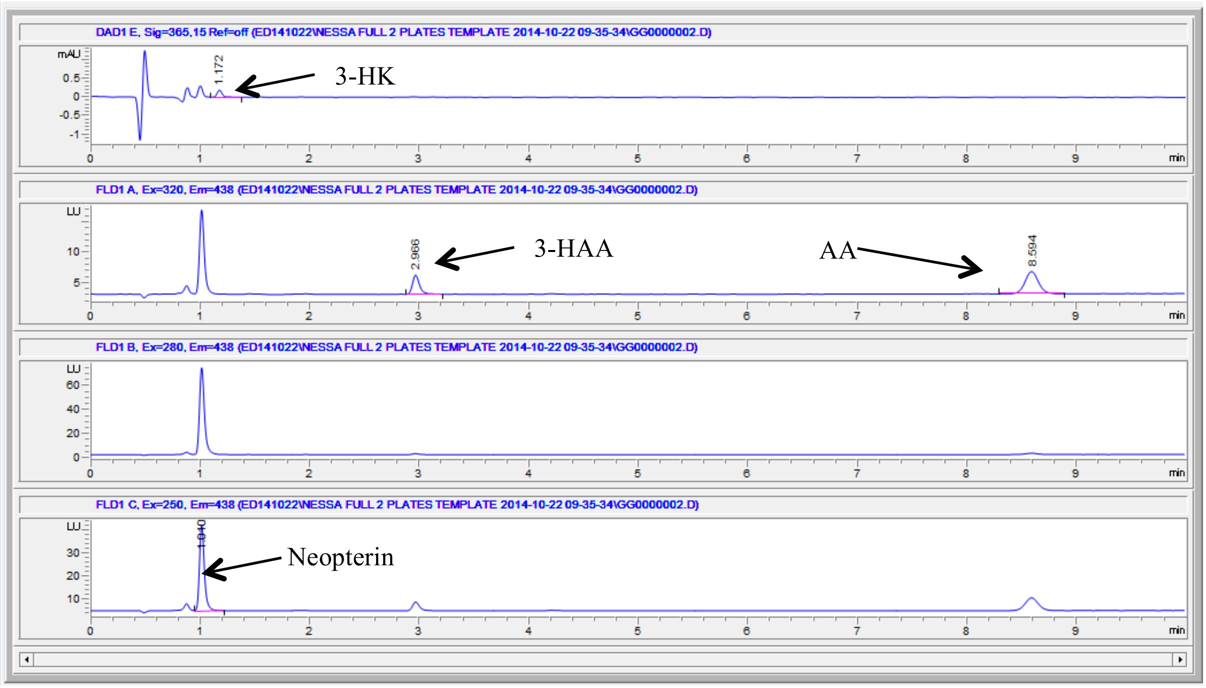


**B.**

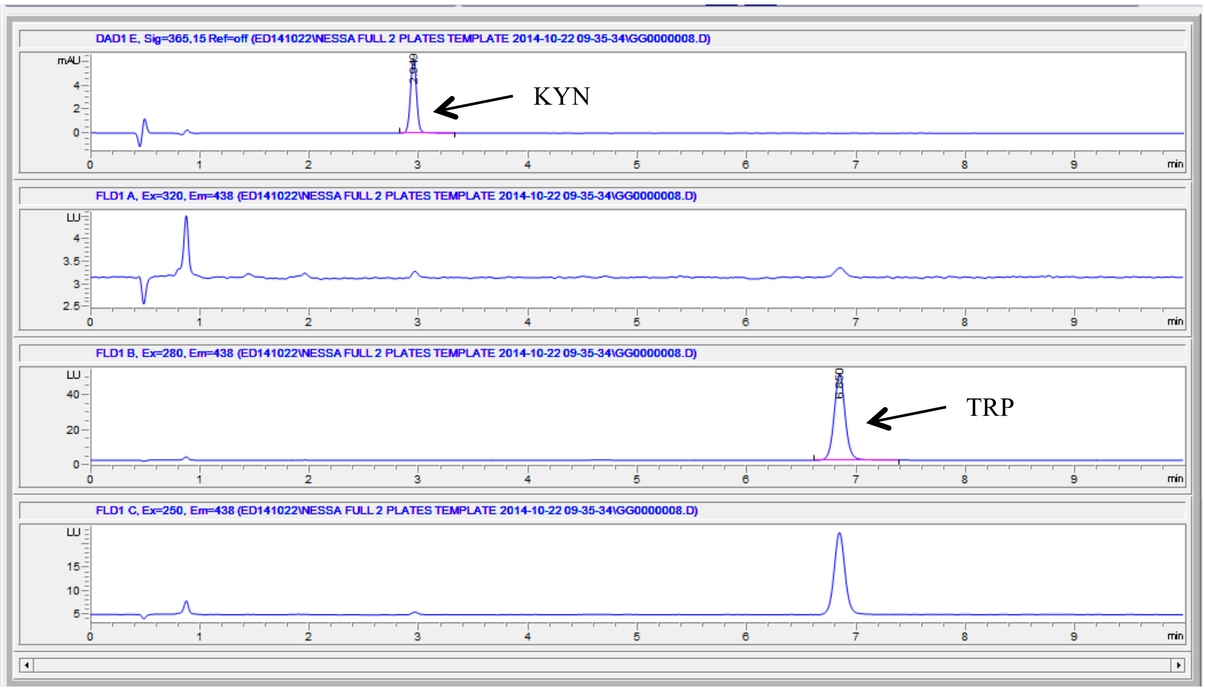

C.

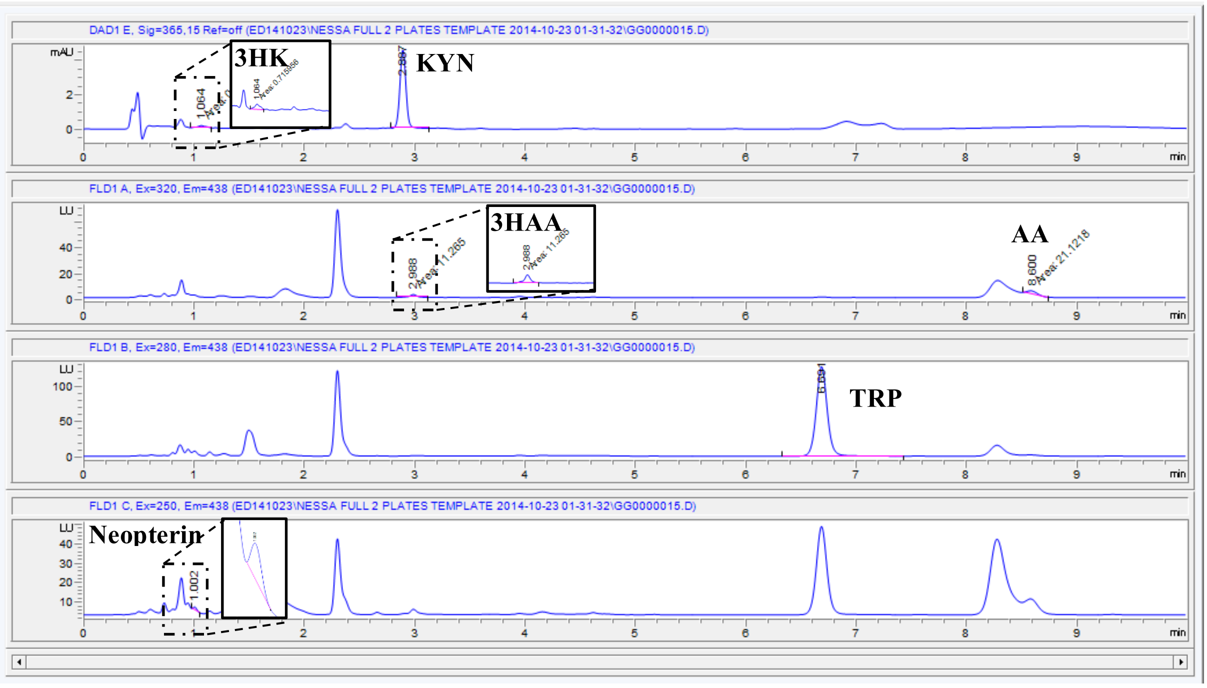

DAD1 E, Sig=365,15 Ref=off (ED141023\NESSA FULL 2 PLATES TEMPLATE 2014-10-23 01-31-32\GG0000015.D)
mAU
3HK
KYN
FLD1 A, Ex=320, Em=438 (ED141023\NESSA FULL 2 PLATES TEMPLATE 2014-10-23 01-31-32\GG0000015.D)
LU
3HAA
AA
FLD1 B, Ex=280, Em=438 (ED141023\NESSA FULL 2 PLATES TEMPLATE 2014-10-23 01-31-32\GG0000015.D)
LU
TRP
FLD1 C, Ex=250, Em=438 (ED141023\NESSA FULL 2 PLATES TEMPLATE 2014-10-23 01-31-32\GG0000015.D)
LU
Neopterin
min

**D.**

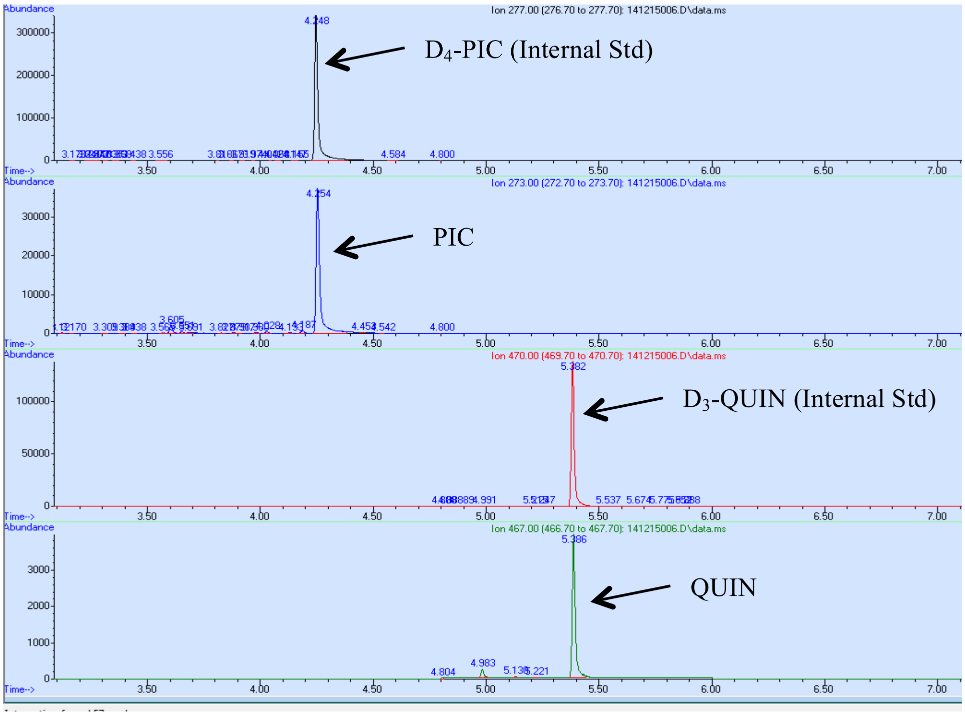

Abundance
Ion 277.00 (276.70 to 277.70): 141215006.D\data.ms
D4-PIC (Internal Std)
Ion 273.00 (272.70 to 273.70): 141215006.D\data.ms
PIC
Ion 470.00 (469.70 to 470.70): 141215006.D\data.ms
D3-QUIN (Internal Std)
Ion 467.00 (466.70 to 467.70): 141215006.D\data.ms
QUIN
Time-->


**E.**

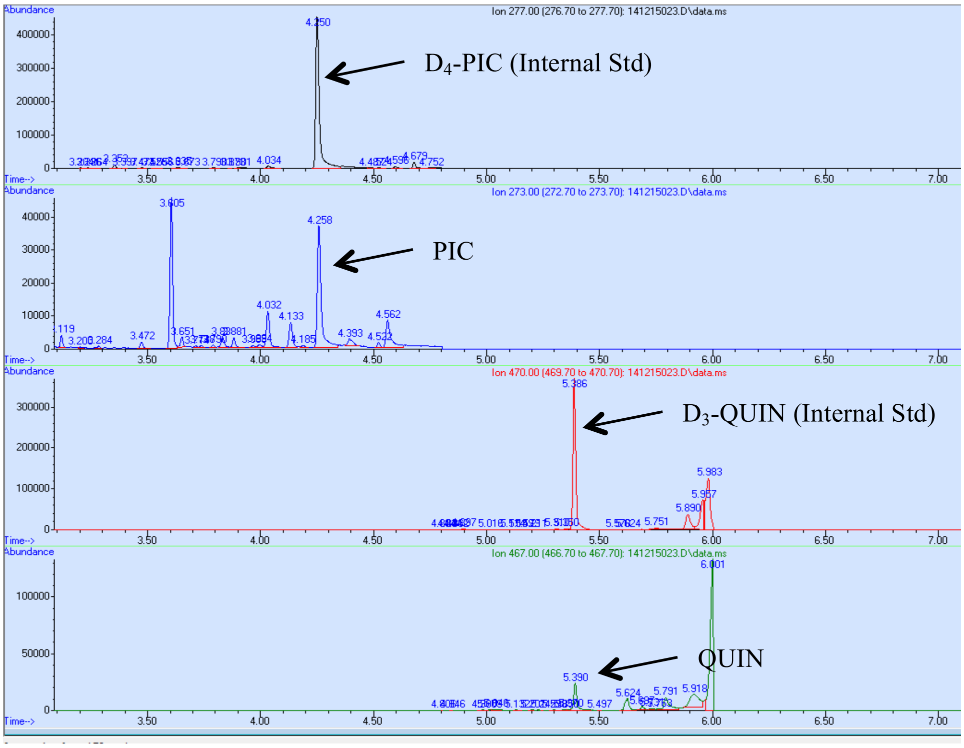

Abundance
Ion 277.00 (276.70 to 277.70): 141215023.D\data.ms
D4-PIC (Internal Std)
Ion 273.00 (272.70 to 273.70): 141215023.D\data.ms
PIC
Ion 470.00 (469.70 to 470.70): 141215023.D\data.ms
D3-QUIN (Internal Std)
Ion 467.00 (466.70 to 467.70): 141215023.D\data.ms
QUIN
Time-->
